# Revealing the Nanoindentation-Induced Plastic Deformation Mechanisms of 4H-SiC: Combined Insights from Molecular Dynamics Simulations and Experiments

**DOI:** 10.3390/ma19153306

**Published:** 2026-08-04

**Authors:** Wuqing Lin, Hongyang Li, Zhongwei Hu, Fuxin Peng, Zhihao Zhou, Yiqing Yu, Yueqin Wu, Xipeng Xu

**Affiliations:** 1 Institute of Manufacturing Engineering, Huaqiao University, Xiamen 361021, China; vincelin37@163.com (W.L.); 18250016105@163.com (F.P.);; 2 State Key Laboratory for High Performance Tools, Huaqiao University, Xiamen 361021, China; 3 Hunan Sanan Semiconductor Co., Ltd., Changsha 410100, China; 4 Fujian Jing’an Optoelectronics Co., Ltd., Quanzhou 362411, China

**Keywords:** 4H-SiC, deformation mechanism, molecular dynamics, basal slip, prismatic slip

## Abstract

4H-SiC substrate is widely employed in semiconductor device fabrication owing to its unique crystal structure and excellent physicochemical properties. However, its deformation behavior during substrate processing remains complex and not fully understood. In this study, molecular dynamics (MD) simulations were performed to systematically investigate the deformation mechanism of single-crystal 4H-SiC during nanoindentation, serving as an atomic-scale analogue of the workpiece–abrasive interaction during substrate processing. Specifically, the relationship between the fluctuations of the load–depth curve and slip were revealed, with particular emphasis on the basal slip (BS) and prismatic slip (PS) behaviors. The results indicate that the fluctuations in the load–depth curve are primarily associated with the nucleation and propagation of dislocations. The initiation of BS is predominantly influenced by the atomic arrangement and stress distribution, while PS initiates at the terminus of BS as the indentation depth increases. Finally, nanoindentation experiments were conducted to validate the reliability of the MD simulations. These findings provide valuable insights into the deformation mechanism and mechanical behavior of 4H-SiC during practical substrate processing.

## 1. Introduction

4H-SiC has been extensively utilized in critical fields, including aerospace, medical devices, and electronic power systems [1]. Consequently, SiC substrates with high structural integrity are required for device-making. Due to its unique crystallographic structure, 4H-SiC exhibits extremely high hardness and brittleness, making it highly susceptible to surface and subsurface damage during substrate manufacturing process. Therefore, a comprehensive understanding of the underlying deformation mechanisms during the machining process is of great practical significance for achieving high-quality, high-efficiency, and high-precision processing of 4H-SiC.

In recent years, advancements in simulation technologies have enabled researchers to gain deeper insights into the deformation behavior of semiconductor materials during various processing techniques [2,3,4,5]. Among these, ultra-precision machining of 4H-SiC has emerged as a prominent area of research and industrial interest [6,7,8,9,10]. In this context, accurate characterization of its deformation mechanisms at the micro and nano scale is essential for improving processing quality.

Nanoindentation, as a critical technique for evaluating the mechanical properties and deformation mechanisms of materials [11,12,13,14], offers valuable insights for addressing key challenges related to material removal and processing performance [15,16,17]. However, due to the limitations of conventional experimental techniques in capturing atomic-scale removal phenomena, molecular dynamics (MD) simulations have been increasingly employed to investigate the atomic-level deformation mechanisms of materials during the machining process [18,19,20,21,22,23]. Numerous studies have used MD simulations to explore the indentation-induced deformation behavior of 4H-SiC. For instance, Zhu et al. [24] investigated the deformation behavior and phase transitions of 4H-SiC under nanoindentation, revealing that basal dislocations predominantly form on the (0001) plane and the indentation process induces lattice distortion. Notably, a phase transition from 4H-SiC to 3C-SiC was observed for the first time, with 3C-SiC grains forming at shallow depths and growing with increased indentation depth. Matsumoto et al. [25] also studied phase transitions in single-crystal 4H-SiC under various nanoindentation conditions and found distinct Raman spectral features depending on crystal orientation. Transmission electron microscopy (TEM) revealed deep subsurface damage zones, including basal dislocations, grain rotation, and microcracks. Liu et al. [26] examined the anisotropic mechanical properties of 4H-SiC wafers using nanoindentation, reporting differences in hardness and fracture toughness between the C-face and Si-face. The C-face exhibited higher hardness, while the Si-face demonstrated greater fracture toughness, attributed to differences in basal plane dislocation nucleation and slip behavior. Their work also highlighted anisotropy in hardness and elastic modulus across different crystallographic directions, emphasizing implications for optimized machining strategies. Shi et al. [27] further investigated the anisotropy of 4H-SiC in various crystallographic planes and orientations using nanoindentation and scratching experiments. Their results revealed periodic variations in maximum lateral force, scratch residual depth, and crack width across different crystallographic directions. They concluded that the C-face yielded superior surface quality in nanoscale abrasive machining, despite more difficult material removal. Chai et al. [28] investigated the mechanical response of single-crystal 4H-SiC at the micro/nano scale and proposed theoretical models for critical indentation depth and load based on fracture strength and contact mechanics. Their nanoindentation tests using a Berkovich indenter revealed that the deformation stages (elastic, plastic, and brittle fracture) could be clearly identified from load–depth curves, with plasticity initiating at a ~10 nm indentation depth. They also analyzed the cracking mechanisms and demonstrated good agreement between experimental and theoretical results regarding the critical indentation depth and load at the pop-in point. Other studies have explored the effects of temperature [29,30], doping [31,32], and ion implantation [33,34] on the indentation-induced deformation behavior of 4H-SiC. In summary, recent studies have focused on mechanical response during the indentation process, deformation forms under mechanical action, and phase transitions. However, the deformation and material removal mechanisms of 4H-SiC during processing remain highly complex [35,36,37,38]. In particular, the correlation between the slip mechanism and mechanical response of 4H-SiC during indentation has not been fully clarified. Therefore, it is of great research significance to further explore the deformation mechanism of 4H-SiC during processing.

The present study employs MD simulations of nanoindentation to investigate the complex deformation mechanisms of 4H-SiC. The simulations elucidate the relationships between slip behaviors and responses of the load–depth curve, the correlation between slip phenomena and atomic structure, and the interactions among crystal slip systems. Complementary nanoindentation experiments were conducted to validate and interpret the simulation results. These findings provide some insights into the deformation and damage mechanisms of 4H-SiC during mechanical processing.

## 2. Simulation and Experimental Details

### 2.1. MD Simulation of Nanoindentation

**Figure 1:**
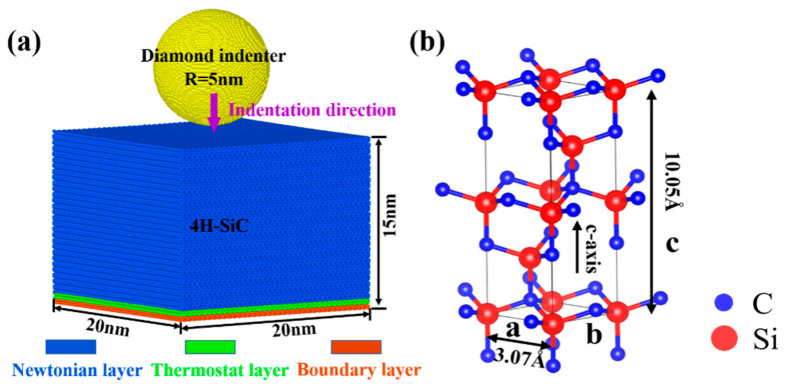
(**a**) Nanoindentation simulation model, (**b**) schematic diagram of the atomic structure of 4H-SiC. a, b, and c correspond to the lattice constants of 4H-SiC in (**b**) and a = b.

LAMMPS (Large-scale Atomic/Molecular Massively Parallel Simulator, Version 2 Aug 2023) is a classical MD simulation software that has been widely used for simulating indentation and scratching processes in semiconductor materials [2,39]. In this study, nanoindentation simulations were carried out using LAMMPS, and the model configuration is illustrated in Figure 1 along with a schematic diagram of the atomic structure of 4H-SiC. The simulation system consists of a diamond indenter and a single-crystal 4H-SiC specimen. The workpiece atoms are divided into three layers from bottom to top: a boundary layer, a thermostat layer, and a Newtonian layer. Atoms in the Newtonian and thermostat layers follow the classical Newtonian mechanics, with their trajectories computed via Newton’s equations of motion. For atoms in the Newtonian layer, their motion—governed by Newton’s second law—is numerically integrated using the Velocity-Verlet algorithm. The dimensions of the workpiece are 20 × 20 × 15 nm^3^, containing a total of 580,125 atoms. The crystallographic orientations along the X, Y, and Z directions are $\left[11\bar{2}0\right]$, $\left[1\bar{1}00\right]$, and [0001], respectively. The present model assumes an ideal crystallographic orientation and does not account for the minor misorientation deviations present in the actual sample. In the simulation, the diamond indenter is treated as a rigid sphere with a radius of 5 nm. Nanoindentation simulations were performed on the basal plane (0001) with the loading direction aligned along the $\left[000\bar{1}\right]$ direction. The model was relaxed under a canonical ensemble (NVT) at a temperature maintained at 297 K, while atoms in the Newtonian layer were simulated under the microcanonical ensemble (NVE). The indentation velocity was set to 50 m/s, with a time step of 0.001 ps and a maximum indentation depth of 5 nm. Periodic boundary conditions were applied along the X and Y directions, while a shrink-wrapped boundary condition was applied in the Z direction. Post-processing and visualization of the simulation results were conducted using OVITO (Open Visualization Tool, Version 3.15.0) [40]. In this study, the Dislocation Extraction Algorithm (DXA) implemented in OVITO was employed to identify and quantify the dislocation line length. The Atomic Strain modifier was utilized to compute the local strain components by evaluating the displacement gradient tensor of each atom relative to its neighboring atoms. Furthermore, based on the local stress deviator of each atom calculated by LAMMPS, the von Mises stress was directly computed and visualized within OVITO.

The selection of an appropriate interatomic potential function is crucial for ensuring the accuracy of atomic-scale simulations. In this study, the simulated system comprises 4H-SiC, which consists of both carbon and silicon atoms, and a diamond indenter composed of carbon atoms. Among the various potentials commonly used in the existing MD simulations of silicon carbide, the Tersoff potential is one of the most widely used [9]. Originally proposed by Tersoff and colleagues in 1989, this potential was designed to describe the atomic bond order interactions between carbon and silicon atoms. However, developments in MD techniques have demonstrated that this potential lacks the precision necessary to accurately describe the atomic interactions within the SiC lattice or the graphitization behavior of diamond. To address these limitations, in 2005, Erhart and Albe et al. improved the Tersoff potential, known as the analytic bond-order potential (ABOP), which exhibits improved descriptions of elastic, defect-related, and thermal properties [9,41]. In this study, the ABOP was selected to describe the atomic interactions within the simulation system.

Furthermore, it should be noted that although the indentation speed and depth used in this simulation differ significantly from those in conventional experiments, the core objective of the simulation is to reveal that the alternating shear resistance determined by the stacking structure governs the initial dislocation nucleation and the competition among slip systems. This originates from the intrinsic crystal structure of the material and the anisotropy of its slip systems—a physical essence that remains robust across a wide range of strain rates. Therefore, the qualitative physical mechanisms underlying crystal slip, stress fluctuations, and damage nucleation, as revealed by the simulation under conditions beyond conventional experimental indentation speeds, remain valid.

### 2.2. Nanoindentation Experiment

To verify the reliability of the MD simulation results, nanoindentation tests were conducted on the Si face of the 4H-SiC sample. The experiments employed a diamond Berkovich indenter operated in a continuous stiffness measurement mode, with a fixed maximum indentation depth of 300 nm [42,43]. Nanoindentation-induced subsurface damage was evaluated using a combined “focused ion beam (FIB) + TEM” method. The surface morphology was examined using a field emission scanning electron microscope (FE-SEM, SU5000) (Hitachi High-Technologies Corporation, Tokyo, Japan), and TEM lamella preparation was conducted at the nanoindentation site via FIB milling. Subsequently, the subsurface damage was detected and analyzed using TEM. It should be noted that there is an inherent geometric difference between the spherical indenter employed in the molecular dynamics simulations and the Berkovich indenter used in the experiments. However, under shallow indentation depths, the tip region of the Berkovich indenter can be reasonably approximated as a sphere with a finite radius of curvature, rendering its contact behavior highly similar to that of an ideal spherical indenter. Therefore, the experimental results presented herein are intended to provide qualitative corroboration of the slip evolution sequence revealed by the simulations rather than to serve as a quantitative comparison.

## 3. Results and Discussions

### 3.1. Load–Depth Curve and Dislocation Analysis

**Figure 2:**
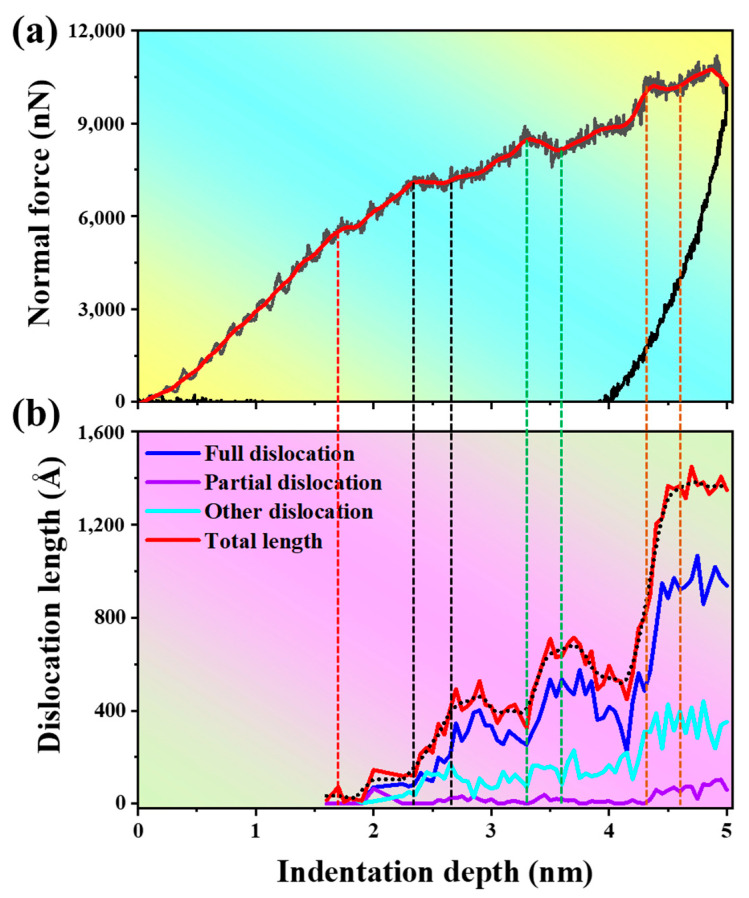
(**a**) Load–depth curve during the simulated indentation process, (**b**) statistical results of lengths of different types of dislocation lines during the indentation process.

Figure 2a shows the load–depth curve obtained from the nanoindentation simulation on the 4H-SiC (0001) substrate, while Figure 2b presents the evolution of dislocation line lengths during the indentation process. A distinct “pop-in” event is observed at an indentation depth of approximately 1.7 nm, signifying the transition from elastic to plastic deformation. Analysis of the dislocation line lengths indicates that this critical point corresponds to the onset of dislocation nucleation.

**Figure 3:**
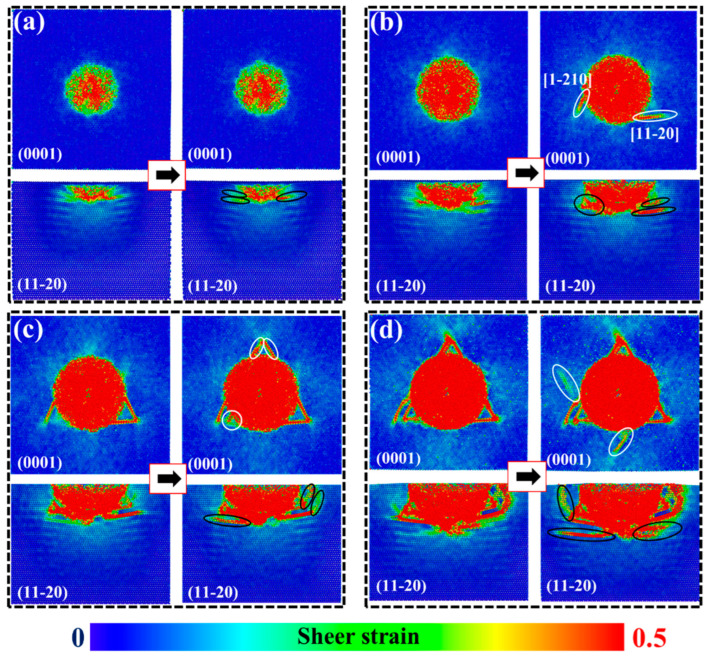
Shear deformation at different stages of the indentation process on the (0001) plane. (**a**) Top view and front view before and after the “pop-in” event, (**b**) top view and front view before and after stage 1, (**c**) top view and front view before and after stage 2, and (**d**) top view and front view before and after stage 3.

Based on the shear strain distributions during the indentation process shown in Figure 3, the shear strain characteristics and slip evolution at the critical point of the “pop-in” event and during the subsequent three stages of plastic deformation are revealed: Figure 3a depicts the shear strain state immediately before and after the “pop-in” event. At this stage, a small amount of BS can be observed within the material, as marked by the black ellipses in Figure 3a, while PS is absent. When the “pop-in” event occurs, a slight increase in the total dislocation line length is detected, as confirmed by the change in dislocation line length shown in Figure 2. Figure 3b corresponds to stage 1 of plastic deformation. At this stage, PS begins to emerge (highlighted by the white elliptical region), while more obvious BS develops on the basal plane (black elliptical region). Figure 3c shows the shear strain characteristics of stage 2, where both BS and PS become more pronounced. It is noteworthy that PS is activated at the terminus of BS, as shown by the white and black elliptical regions. In stage 3, as shown in Figure 3d, BS and PS continue to intensify and expand as the indentation depth increases. These observations suggest that continuous slip activity induced by sustained external loading is a key factor contributing to the fluctuations observed in the load–depth curve during the nanoindentation process. The interplay between BS and PS systems thus plays a critical role in the evolution of plastic deformation in 4H-SiC. Under indentation loading, localized stress concentration first induces the nucleation of basal slip dislocations. Accompanied by lattice distortion, high-energy prismatic slip systems are activated, causing redistribution of the stress field. The synergy and competition within this multi-slip process directly lead to significant fluctuations in the load–depth curve.

### 3.2. The Activation Mechanisms of BS and PS

**Figure 4:**
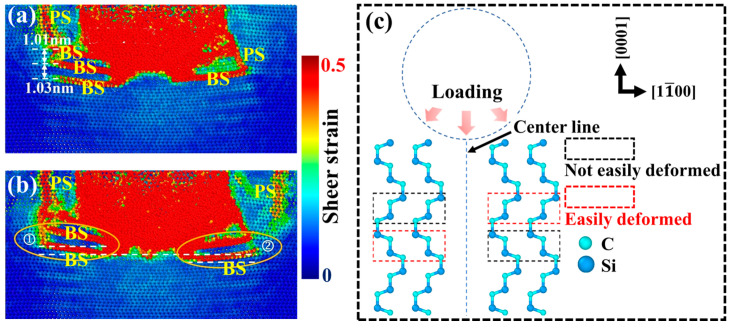
Shear deformation at varying indentation depths on the (0001) plane. Front view at an indentation depth of (**a**) 4 nm and (**b**) 4.5 nm, (**c**) schematic representation of the loading capacity of different atomic regions under indentation.

As shown in Figure 4a, both the PS and BS are activated at an indentation depth of 4 nm. The spacing between the BS bands exhibits a regular periodicity, with vertical spacing between adjacent slip bands approximately 1.01 nm and 1.03 nm—values very close to the lattice constant c (1.005 nm) of 4H-SiC. Figure 4b illustrates that BS primarily occurs underneath both sides of the indentation zone. As the indentation depth increases, an alternating slip behavior is observed on both sides, with a vertical spacing along the c-axis direction equivalent to half a stacking cycle (c/2). This alternating pattern originates from differences in bearing resistance among atomic layers within the stacking sequence.

Figure 4c presents a schematic of the atomic bonding configuration on the 4H-SiC $\left(11\bar{2}0\right)$ plane, used to analyze the local atomic stress distributions. During loading, different regions exhibit distinct deformation patterns. The region directly beneath the indenter experiences the maximum normal load, resulting in atomic bond breakage, crystal structure disordering, and partial amorphization. The surrounding indentation region is mainly affected by shear stress, promoting crystal slip. However, due to the inherent crystal symmetry and periodic structure of 4H-SiC, the ease of slip varies significantly among different crystallographic orientations. Atoms within the black boxed region are highly compressed, leading to reduced interatomic spacing and increased interatomic forces, which hinder slip initiation. On the contrary, atoms in the red boxed region experience tensile stress, where the increased atomic spacing reduces the interatomic force, facilitating slip. Consequently, this region becomes the preferred nucleation site for BS. This indicates that the ABCA stacking sequence of 4H-SiC leads to a periodic fluctuation in the interlayer shear modulus, resulting in alternating high and low shear resistance during dislocation glide, which significantly affects its plastic deformation and fracture behavior.

**Figure 5:**
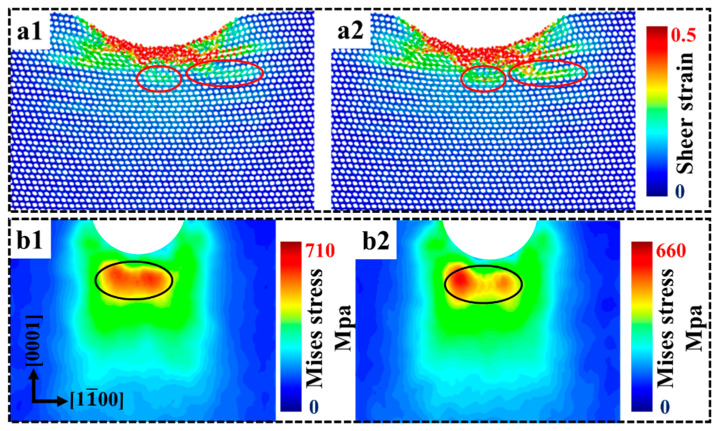
Distribution of shear strain and von Mises stress before and after slip. Shear strain at indentation depth of (**a1**) 1.5 nm and (**a2**) 1.6 nm, von Mises stress at indentation depth of (**b1**) 1.5 nm and (**b2**) 1.6 nm.

Figure 5 shows the shear strain distribution and the corresponding von Mises stress distribution before and after slip initiation. By comparing the shear strain fields at indentation depths of 1.5 nm (Figure 5(a1)) and 1.6 nm (Figure 5(a2)), it can be observed that an increase in indentation depth leads to further slip propagation in the lower right region of the indentation zone (red elliptical area). The results indicates that at an indentation depth of 1.5 nm, the material already exhibits a tendency of BS. When the indentation depth increases to 1.6 nm, BS behavior becomes more pronounced (red elliptical region), accompanied by the nucleation of a small number of dislocations.

The corresponding von Mises stress distributions are presented in Figure 5(b1,b2), respectively. Before slip initiation (indentation depth of 1.5 nm), a significant stress concentration exists directly beneath the indenter (black elliptical region), as illustrated in Figure 5(b1). After slip occurs (indentation depth of 1.6 nm), the von Mises stress magnitude in this region decreases, and the size of the high-stress area is noticeably reduced, as shown in Figure 5(b2), indicating that the slip process effectively relieves the locally accumulated stresses. It is worth noting that the stress distribution pattern undergoes a marked change at this stage: although stress concentration still exists beneath the indenter, the high-stress regions shift laterally toward to both sides of the indentation. This dynamic evolution of stress distribution further confirms the strong relationship between the hierarchical slip deformation mechanism of 4H-SiC and the local stress state during indentation.

**Figure 6:**
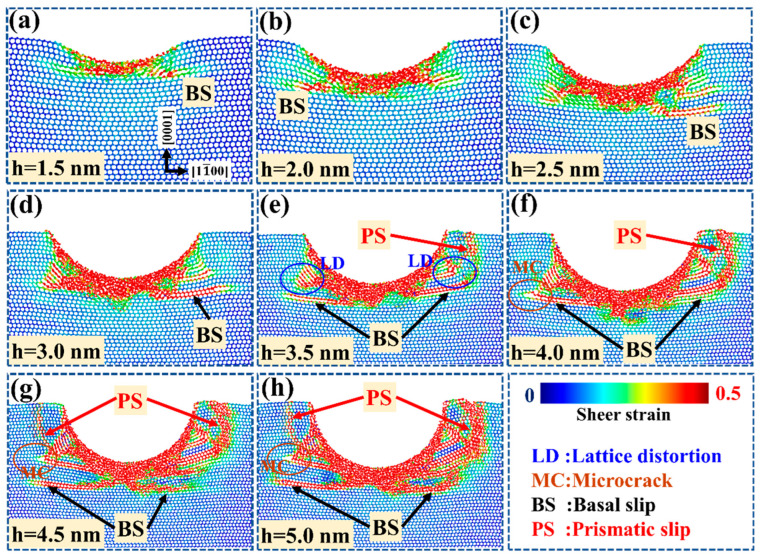
Crystal slip evolution during the nanoindentation process. The indentation depth corresponding to (**a**–**h**) is 1.5–5.0 nm.

Figure 6 systematically illustrates the evolution of BS and PS as the indentation depths increases from 1.5 nm to 5.0 nm. During the initial loading phase (Figure 6a–d), BS is the first to be activated. The increased indentation depth promotes BS and induces pronounced lattice distortion at its termination. Stress concentrations develop in these distorted regions, leading to the formation of microcracks near the lattice distortion zones. The emergence of these microcracks hinders further propagation of BS and subsequently activates PS (Figure 6e–h). These observations clearly demonstrate a strong sequentiality between PS and BS in the deformation of 4H-SiC during nanoindentation. These findings provide critical atomic-scale evidence for elucidating the plastic deformation mechanism governing the material’s response under mechanical loading.

### 3.3. Nanoindentation Experimental Validation

The primary objective of the experiments is to qualitatively validate the sequential activation order of BS followed by PS as well as the spatial distribution characteristics of slip bands and amorphous regions observed in the molecular dynamics simulations. Given the discrepancies between simulations and experiments in terms of indenter geometry, scale, and loading rate, this study focuses on the qualitative consistency of the deformation mechanisms rather than on quantitative agreement.

**Figure 7:**
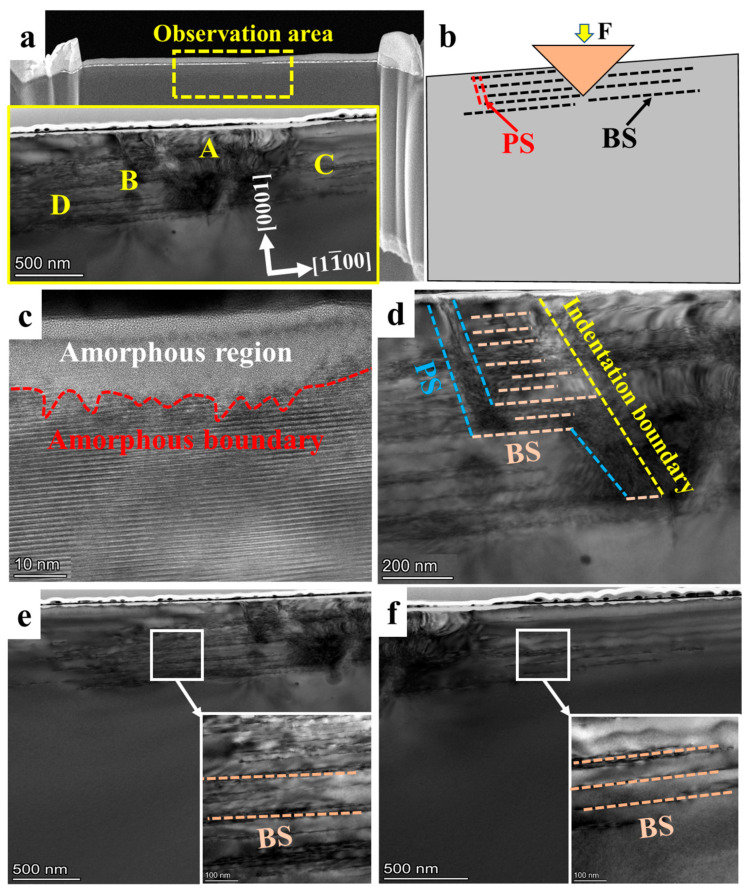
TEM images of the indentation morphology. (**a**) Overall subsurface damage morphology, (**b**) schematic representation of subsurface damage forms, (**c**–**f**) enlarged view of regions A, B, C and D.

Figure 7a presents an SEM image of the indentation region and the inset TEM image of overall subsurface damage morphology, with labeled areas enlarged for detailed analysis. A schematic representation of the subsurface damage is provided in Figure 7b. A high-magnification TEM image of region a (Figure 7c) reveals a distinct amorphous transformation zone directly beneath the indenter. This amorphization is attributed to the extremely high contact stress imposed by the indenter during nanoindentation, which triggers an amorphous phase transformation once the local stress exceeds the bond strength of 4H-SiC crystals [24,25]. As shown in Figure 7d, TEM analysis of region b indicates the coexistence of BS and PS on both sides of the indentation. Notably, the propagation of BS is significantly hindered in the regions behind where PS was activated. These phenomena observed in the TEM images are highly consistent with the MD simulation results: following the formation of the amorphous zone, slip preferentially initiates along the close-packed (0001) basal plane to release accumulated strain energy. The distribution characteristics of BS are closely related to the layer sequence of 4H-SiC, manifesting as parallel banded structures with regular spacing. This indicates that the critical condition for slip system activation is governed by the local stress level and the critical resolved shear stress. The experimental transmission electron microscopy images indeed reveal a slip sequence consistent with the molecular dynamics simulations over a wide range of indentation depths, which in turn supports the scale robustness of the simulated mechanisms.

Figure 7e,f show that region c and region d are dominated by high-density BS. Notably, the BS density on the left side of the indenter (region d) is significantly higher than that on the right side of the indenter (region c). This asymmetry arises because the (0001) plane is slightly tilted (~2.3°) relative to the horizontal plane. The indenter is not perpendicular to the (0001) plane, causing the indenter to exert greater shear stress on the left side during loading. As a consequence, the left side exhibits more intensive BS and PS activity. This asymmetric slip distribution reflects the influence of the nonuniform stress field caused by the indenter geometry and loading direction. The experimental results align well with the MD simulations, collectively elucidating the damage evolution mechanism of 4H-SiC at the nano scale. These insights provide valuable theoretical support for optimizing the abrasive machining processes, especially in controlling subsurface damage depth in 4H-SiC.

## 4. Conclusions

In this study, the deformation mechanisms of 4H-SiC during nanoindentation were investigated through MD simulations and indentation experiments. The relationship between the load–depth curves and material slip behavior during the indentation process and the interaction between BS and PS were systematically analyzed. The main conclusions are as follows:

Under indentation loading, local stress concentration first induces the nucleation of BS. Accompanied by lattice distortion, PS is activated, leading to a redistribution of the stress field. The competition in the multi-slip process not only causes significant fluctuations in the load–depth curve but also directly manifests as characteristic “pop-in” events on the curve. These events correspond to the initiation of dislocation nucleation, with slip occurring along specific slip systems and accompanied by dislocation generation and stress release, thereby forming regular fluctuations in the macroscopic curve.

The occurrence of BS is closely related to atomic arrangement and stress distribution. The ABCA stacking sequence of 4H-SiC leads to periodic fluctuations in the interlayer shear modulus, generating alternating high and low shear resistance during dislocation slip, which significantly influences its plastic deformation and fracture behavior. The periodicity of BS bands aligns with the lattice constant and stress concentration under deeper indentation, thereby promoting the activation of BS.

In nanoindentation simulations, a close relationship exists between BS and PS. BS is activated prior to PS, and its accumulation induces lattice distortion at its termini. As the indentation depth increases, PS becomes activated and subsequently hinders the further propagation of BS.

TEM images reveal the direct formation of an amorphous structure beneath the indentation, flanked by extensive BS. The activation of PS occurs after BS and further impedes its propagation, a finding consistent with molecular dynamics simulations. Additionally, the non-perpendicular alignment of the loading direction with the (0001) surface, combined with the indenter’s geometry, leads to an asymmetric distribution of BS.

This study only considers the loading condition along the direction normal to the (0001) plane. Given the strong mechanical anisotropy of 4H-SiC, future work is warranted to systematically investigate the activation sequence and competitive mechanisms between basal slip (BS) and prismatic slip (PS) under different crystallographic planes (e.g., m-plane, a-plane) and various in-plane loading directions in order to achieve a comprehensive understanding of its plastic deformation behavior.

## Data Availability

The original contributions presented in this study are included in the article. Further inquiries can be directed to the corresponding author.
